# Recent Advances in Carcinogenesis Transcription Factors: Biomarkers and Targeted Therapies

**DOI:** 10.3390/cancers15194673

**Published:** 2023-09-22

**Authors:** Ann M. Bode, Tianshun Zhang

**Affiliations:** The Hormel Institute, University of Minnesota, Austin, MN 55912, USA

Carcinogenesis, the process by which normal cells transform into cancer cells, is complex and multifaceted. Understanding the molecular mechanisms underlying this multitude of diseases is crucial for the development of effective diagnostic tools and targeted therapies. Transcription factors (TFs) play a central role in regulating gene expression and have emerged as key players in the development and progression of various cancers. In simple terms, a transcription factor (i.e., sequence-specific DNA-binding factor) is a specialized protein that binds to DNA and governs the rate of transcription of genetic information from DNA to messenger RNA [1,2]. Because of their governing ability, TFs possess the unique capacity to either activate or inhibit the expression of specific genes, making them pivotal regulators of diverse cellular processes.

In the context of carcinogenesis, the aberrant activation or inactivation of TFs can disrupt the delicate balance of gene expression, leading to uncontrolled cell growth and the formation of tumors. Recent research has underscored the significance of distinct transcription factors in various cancer types, shedding light on their roles and potential therapeutic implications. In this Special Issue of *Cancers*, five articles highlight the important role of certain transcription factors in carcinogenesis.

Two of the articles, one a review [3] and the other a research article [4], elaborate on the role of the sex-determining region Y (SRY)-box 1 (SOX) family proteins in cancer. In the review, authors focus on the role of the SOX family of transcription factors in the tumor immune microenvironment (TIME) of hepatocellular carcinoma (HCC), a potentially deadly liver cancer. Authors provide an overview of the structure, classification, and physiological function of SOX family members. They concentrate primarily on the TIME in HCC and the association between SOX family members and various immune components. In addition, they discuss the opportunities and challenges of targeting the SOX family in cancer.

As the authors indicate, the TIME is a highly complex system comprising both innate and adaptive immune cells that play a central role in the development and progression of cancer, including HCC [5]. Based on the literature, authors contend that the SOX family members play an important role in remodeling the TIME due to their impact on different immune cells. In particular, SOX family members greatly influence type 1 interferon signaling in various cancers and authors provide detailed evidence from the literature. They performed an intricate analysis and show that SOX family members maintain a significant and comprehensive positive correlation with certain immunosuppressive cells (e.g., tumor-associated macrophages, regulatory T cells, neutrophils, and regulatory B cells) in HCC.

Overall, this is a comprehensive and informative review focusing on HCC and the tumor immune microenvironment. Within this complex microenvironment, the SOX transcription factors assume a crucial role, orchestrating gene expression and influencing a myriad of processes both within the tumor and its surrounding tissue. Among these factors, SOX transcription factors are emerging as key regulators of the tumor immune microenvironment, wielding their influence over immune cells, stromal cells, and the overall tumor microenvironmental landscape. This newfound understanding positions SOX transcription factors as promising targets for cancer research and potential therapeutic interventions aimed at enhancing the efficacy of cancer treatments [3].

The research article focusing on SOX published in this Special Issue delves into the enigmatic role of SOX1 in lung cancer, unearthing its robust potential as a tumor suppressor through its effective blocking of the hairy and enhancer of split 1 (HES1) [4]. The HES1 is known to be associated with various processes that facilitate cancer metastasis [6,7,8,9,10]. The *SOX* family genes encode a group of proteins that share a similar DNA-binding high-mobility-group (HMG) domain [11] and are responsible for regulating diverse signaling pathways in cancers comprising either tumor suppressor genes [12,13,14,15] or oncogenes [16,17,18]. The authors of the current study [4] previously showed that *SOX1* is hypermethylated in various cancers [19,20,21], including non-small-cell lung cancer (NSCLC). They also showed that SOX1 can act as a tumor suppressor by interfering with Wnt/β-catenin signaling in HCC [21] and cervical cancer [22].

In the current study [4], they confirm that hypermethylation of *SOX1* in lung cancer does indeed contribute to *SOX1* silencing or downregulation. Furthermore, the restoration of expression of SOX1 in cells or mice suppresses lung cancer growth and invasion. Additional experiments exploring possible mechanisms for these effects show that SOX1 directly targets the *HES1* promoter to reduce its expression. The downregulation of HES1 was confirmed to contribute to the effectiveness of SOX1 suppressive effects against lung cancer cell growth. In conclusion, researchers show that SOX1 performs its tumor suppressor function by directly binding to the *HES1* promoter and thus *SOX1* may be a critical tumor suppressor gene in lung cancer. These findings [4] not only enhance our understanding of lung cancer’s intricacies but also open up novel avenues for therapeutic intervention.

A second review article [23] in this Special Issue focuses on targeting transcription factors (TFs) as biomarkers in the epithelial–mesenchymal transition (EMT), a pivotal process in malignant progression [24,25,26]. In general, the EMT characteristically exhibits a loss of intercellular adhesion and epithelial markers (e.g., E-cadherin and claudins) and a gain of mesenchymal markers (e.g., vimentin and N-cadherin) [27]. Several families of transcription factors, including Myc, the Snail protein, Zinc finger E-box binding (ZEBs) proteins, and Twist proteins are key drivers in controlling the EMT, and their deregulation can trigger the transformation of cancer cells into a more invasive and mobile state by affecting genes involved in cell adhesion, migration, and invasion. In particular, Myc proteins promote the EMT, while Snails and Twists repress cell adhesion molecules like E-cadherin. The ZEBs, on the other hand, suppress E-cadherin and boost mesenchymal markers. These transcription factors contribute to cancer cells breaking away from primary tumors, entering the bloodstream or lymphatic vessels, surviving in circulation, and establishing metastatic sites.

This particular review concentrates on categorizing and listing the transcription factors involved in the EMT based on their various DNA-binding domains (DBDs). These domains include TFs containing DNA-binding basic domains, zinc-coordination DNA-binding domains, helix-turn-helix binding domains, other all-alpha-helical DNA-binding domains, immunoglobulin fold DNA-binding domains, and beta-hairpin exposed by an alpha/beta-scaffold DNA binding domains.

An overview of the epigenetic regulation pathways of TFs involved in the EMT is also presented followed by the therapeutic implications and challenges of targeting EMT-TFs in cancer. Currently no drugs have been approved by the FDA because of the complexity of the EMT. Thus, understanding the roles of various TFs is vital for developing targeted therapies and identifying specific biomarkers for personalized treatments. Overall, this review provides a comprehensive systematic classification of the various types of TFs involved in the EMT process based on their DNA-binding domain structure [23].

Two of the five articles published in this Special Issue are associated with cancers that are difficult to treat due to their complex heterogeneity or their resistance to therapy.

The additional compelling signaling pathways include those mediated through the primary cilium (PC), a long, thick organelle that protrudes from the tip or apex of almost all cell types, particularly epithelial cells. The primary signaling pathway associated with the PC is probably the hedgehog (Hh) pathway. The exact role of primary cilia in transducing Hh signaling is complex and context-dependent because cilia can act as both positive and negative mediators of Hh signaling. Deregulation of Hh signaling has an important role in the development and progression of cancer, especially MPM. In this Special Issue, researchers investigated the presence of the PC in malignant pleural mesothelioma (MPM) [28], a common cancer of the pleura with a poor prognosis because of limited treatment options. This cancer is extremely heterogenic, which contributes to the lack of available therapies. Barbarino et al. found a high diversity in PC expression, with its loss correlating with more aggressive cancer. The GLi1 family of transcription factors that mediate Hh signaling are significantly higher in MPM patients. Importantly, in the current study, researchers offer compelling evidence of PC-independent regulation of the Hh pathway. In fact, their results suggest that loss of the PC is a frequent occurrence in aggressive MPM but does not preclude activation of the Hh pathway canonically or non-canonically. These findings highlight the need for innovative approaches for addressing the patient-specific treatment challenges posed by this formidable, highly heterogenetic cancer [28].

The fifth article discussed in this editorial ties together the EMT and TME because of its focus on hypoxia [29], a hallmark feature of the solid tumor microenvironment characterized by oxygen deprivation and the activation of hypoxia-inducible factors [30,31,32,33]. The estrogen receptor alpha (ERα) is known to be the primary transcription factor driving the majority of hormone-dependent breast cancers. Hypoxia has been reported to suppress ERα expression in breast cancer [34,35,36]. In this report, authors show that hypoxia either suppresses or enhances the activation of a fraction of ERα-responsive genes. The ChIP-seq revealed some unexpected results that led authors to conclude that hypoxia interferes with estrogenic signaling in mediating the efficacy of endocrine therapies.

Specifically, authors verify that hypoxia mainly inhibits ERα protein expression. Loss of ERα expression has been associated with endocrine-resistant breast cancer, although this is somewhat controversial. They also observed that a high expression of HIF1α is associated with poor overall survival in ERα-positive patients, which they conclude that this confirms that when solid breast tumors become hypoxic, overall patient survival is reduced. Based on this study, the role of hypoxia in luminal ERα-positive breast cancer emerges as a critical factor interfering with endocrine therapies. Notably, this association is linked to a less favorable clinical prognosis for breast cancer patients [29].

Recent advances in our understanding of the role of various transcription factors in carcinogenesis have provided new opportunities for the development of innovative diagnostic tools and targeted cancer therapies. The identification of TF biomarkers holds promise for early cancer detection and prognosis, while the development of TF-targeted therapies represents a significant leap forward in personalized cancer treatment. As research in this field continues to progress, we can anticipate more effective strategies for treating cancer and improving patient outcomes.
